# Data for Genetic Analysis Workshop 16 Problem 1, association analysis of rheumatoid arthritis data

**DOI:** 10.1186/1753-6561-3-s7-s2

**Published:** 2009-12-15

**Authors:** Christopher I Amos, Wei Vivien Chen, Michael F Seldin, Elaine F Remmers, Kimberly E Taylor, Lindsey A Criswell, Annette T Lee, Robert M Plenge, Daniel L Kastner, Peter K Gregersen

**Affiliations:** 1 Departments of Epidemiology and Biomathematics, University of Texas, MD Anderson Cancer Center, 1155 Pressler Street, Houston, Texas 77030, USA; 2 Rowe Program of Human Genetics, Department of Medicine, University of California, One Shields Avenue, 4303 Tupper Hall, Davis, California 95616, USA; 3 Genetics and Genomics Branch, National Institute of Arthritis and Musculoskeletal and Skin Diseases, National Institutes of Health, 9000 Rockville Pike, Building 10, Room 6N226, Bethesda, Maryland 20892, USA; 4 The Rosalind Russell Medical Research Center for Arthritis, Department of Medicine, Division of Rheumatology, University of California at San Francisco, 374 Parnassus Avenue, Box 0500, San Francisco, California 94143-0500, USA; 5 Center for Genomics and Human Genetics, North Shore-Feinstein Medical Research Institute, 350 Community Drive, Manhasset, New York 11030, USA; 6 Program in Medical and Population Genetics, Broad Institute of Massachusetts Institute of Technology and Harvard, 320 Charles Street, Cambridge, Massachusetts 02141, USA

## Abstract

For Genetic Analysis Workshop 16 Problem 1, we provided data for genome-wide association analysis of rheumatoid arthritis. Single-nucleotide polymorphism (SNP) genotype data were provided for 868 cases and 1194 controls that had been assayed using an Illumina 550 k platform. In addition, phenotypic data were provided from genotyping DRB1 alleles, which were classified according to the rheumatoid arthritis shared epitope, levels of anti-cyclic citrullinated peptide, and levels of rheumatoid factor IgM. Several questions could be addressed using the data, including analysis of genetic associations using single SNPs or haplotypes, as well as gene-gene and genetic analysis of SNPs for qualitative and quantitative factors.

## Background

Rheumatoid arthritis is a complex disease with a moderately strong genetic component. The recurrence risk ratio for siblings is typically estimated at around 6 in Caucasians, but it has a broad range of values, primarily because the prevalence in the population is not well characterized [1]. The prevalence also varies among populations, ranging from around 0.8% in Caucasians to 10% in some Native American groups. Females are generally at higher risk than males, with about a 3 to 1 predominance of females to males. The mean age of disease onset is in the fifth decade with considerable variability in age at presentation, including occasional presentation in the teenage years.

The HLA region on 6p21 has been implicated by numerous studies, and there is consistent evidence that DR alleles contribute to disease risk. The 'shared epitope' hypothesis was proposed by Gregersen et al. [2] to explain the organization of risk for rheumatoid arthritis from DR alleles. According to this hypothesis, individuals who share a QK/RRAA motif in positions 70 to 74 of the DR molecule show an increased risk for disease. The alleles that confer increased risk for rheumatoid arthritis include DRB1*0101, *0102*, *0104*, *0105*, ***0401***, ***0404***, ***0405***, ***0408***, ***0409***, *1001*, *1402*, and *1406*, with highest risk alleles in bold [3]. This model was not quite sufficient to explain risk according to DR types, and newer models utilizing data from positions 70 to 74 have been developed [4,5]. DR effects on risk for rheumatoid arthritis also show a complex effect on risk for rheumatoid arthritis, but presence of two risk alleles generally increases risk substantially more than the risk associated with heterozygosity for risk and nonrisk alleles. Aside from the main effects of DR, there is also evidence for interactions with other HLA loci or haplotypic effects including the class 1 region and the central MHC [6]. Certain DR alleles, notably DR3 [7,8], can occur on a background of extended linkage disequilibrium, for which the extended haplotype confers increased risk, even though DR3 alleles alone do not increase risk.

Two quantitative phenotypes that are used for identifying rheumatoid arthritis affected individuals include anti-cyclic citrullinated peptide (anti-CCP) and rheumatoid factor IgM autoantibodies. The heritability of these measures is hard to obtain from the selected sib pairs we are studying. After proband correction, the heritability estimates are 11% and 30%, while before correction the heritabilities are 15% and 67%. Specific autoantibodies are noted to co-occur with rheumatoid arthritis. Rheumatoid factor IgM has been correlated with erosive arthritic disease. However, anti-CCP is more specific for the disease and is a better predictor of erosive outcome [9]. Elevations of anti-CCP have been noted to predict increased risk for development of rheumatoid arthritis [10]. The shared-epitope alleles are strongly associated with the presence of anti-CCP antibodies, and there is evidence that this effect is modulated by HLA-DR3 [8].

Alleles at the *PTPN22 *locus have been shown to confer an increased risk for rheumatoid arthritis [11]. At least two alleles of *PTPN22 *have been implicated as causing increased risk for rheumatoid arthritis; the R620W allele in rs2476601 (hCV16021387) confers 1.7- to 1.9-fold increased risk to heterozygotes and higher risks to homozygous carriers. These findings have further been confirmed by analysis of transmission of *PTPN22 *alleles to affected offspring in families [12]. Increased risk has also been noted for either hCV8689108 or hCV25762283 [13], with some indeterminacy because of linkage disequilibrium among these markers (and others in the region).

The *CTLA4 *locus on chromosome 2q33 has been associated with mildly increased risk for rheumatoid arthritis [14]. In addition, alleles at loci in the *TRAF1/C5 *region are associated with rheumatoid arthritis risk [15]. A targeted association study showed that alleles of *STAT4 *[16] are associated with rheumatoid arthritis risk, but these associations are too weak to reach genome-wide levels of association in the data set that we have here provided. Similarly, a locus on chromosome 6q (*TNFAIP3*) that is associated with rheumatoid arthritis risk has relatively weaker effects [15]. Additional loci that have been implicated in Caucasian rheumatoid arthritis populations include *CD40 *(20q13), *PRKCQ *(10p15), and *CCL21 *(9p13), among others [17,18].

Aside from identified genetic factors and sex, few environmental cofactors have been identified as affecting risk for rheumatoid arthritis. However, current smoking confers about a two-fold increased risk [7]. Klareskog et al. [19] showed that the risk from smoking for rheumatoid arthritis is particularly high among individuals who have a shared-epitope allele and who also have elevated levels of anti-CCP antibodies. The biological basis for this rather complex interaction appears to reflect increased citrullination of peptides among smokers, and presentation of citrullinated peptides by shared-epitope alleles.

The data set submitted for the Genetic Analysis Workshop 16 (GAW16) was designed with a primary goal of allowing the identification of genetic factors that predispose to rheumatoid arthritis using association methods. Given some previously identified evidence for effects of smoking on rheumatoid arthritis risk and difference in risk according to sex, there is an interest in identifying gene-environment and gene-gene combinations that yield particularly high risks to individuals for rheumatoid arthritis.

## Methods

The cases that we made available for analysis by participants in GAW16 comprised independent individuals who had met the American College of Rheumatology criteria for rheumatoid arthritis. These cases comprise a single member of 445 sibpairs that were studied as a part of the North American Rheumatoid Arthritic Consortium because they had at least one additional sibling with rheumatoid arthritis, and an additional 423 cases who were not selected for family history. The cases were recruited from across the United States. Cases are predominantly of Northern European origin. The controls, derived from the New York Cancer Project, were enrolled in the New York metropolitan area [20]. These controls are somewhat enriched for individuals of Southern European or Ashkenazi Jewish ancestry compared with cases. Structure across European populations has been described [21,22], and some autoimmune predisposing alleles, such as the *PTPN22 *R620W and *HLA DR4 *alleles show strong clines across European populations. In addition, alleles at other loci such as the Lactase Persistence gene (*LCT*) show strong clines across European populations. Evidence in association studies for an effect of the *LCT *locus on case/control status likely reflects false-positive association due to stratification. Studies within Europe have confirmed the associations of *PTPN22 *and *HLA *but have not confirmed effects of *LCT *on risk for rheumatoid arthritis.

The GAW16 rheumatoid arthritis data is part of ongoing studies to identify genetic associations of rheumatoid arthritis [14]. The data that were provided to GAW16 included results from genotyping 868 cases and 1194 controls after the application of quality control procedures that included removing individuals who had a low overall call rate (<95%) of single-nucleotide polymorphisms (SNPs), removing first degree relatives, and removing duplicated and contaminated samples. The data that were provided as a part of Genetic Analysis Workshop 16 Problem 1 were included in a previous publication [15], which identified the *TRAF1/C5 *locus as contributing to susceptibility to rheumatoid arthritis. This earlier publication included additional data that were not provided to the Genetic Analysis Workshop 16 Problem 1 from a study of early-onset rheumatoid arthritis conducted in Sweden. Aside from the *TRAF1/C5 *locus, there were significant effects from the HLA region and *PTPN22 *that can be readily discerned from the data.

Data that were provided to Genetic Analysis Workshop 16 participants included affection status with rheumatoid arthritis, sex, *DRB1 *alleles detected by serology and further defined using DNA probes for DRB1*04 and DRB1*01 alleles, number of shared epitopes carried, the anti-CCP titer, rheumatoid factor IgM level, and 545,080 genotypes derived from Illumina genotyping arrays. All rheumatoid arthritis cases and 589 controls were genotyped on the HumanHap500 v1, 358 controls were done on the Human Hap500 v3.0, and 247 controls were done on HumanHap300 and HumanHap240 arrays.

## Discussion

Rheumatoid arthritis results from a complex interaction of genetic and environmental factors. Data that were provided for GAW16 were derived from a large number of cases and controls who had been genotyped using dense SNP arrays. These data were sufficient to identify many genetic loci influencing rheumatoid arthritis risk. In addition, we provided data for two autoantibodies that are often elevated among individuals who have rheumatoid arthritis. Aside from identifying genetic factors influencing rheumatoid arthritis, the data that were provided can be used to investigate population structure in European populations, methods for inferring SNPs, and modeling approaches when multiple genetic factors influence disease risk.

## List of abbreviations used

anti-CCP: Anti-cyclic citrullinated peptide; GAW: Genetic Analysis Workshop; SNP: Single-nucleotide polymorphism

## Competing interests

The authors declare that they have no competing interests.

## Authors' contributions

CIA developed data to be transmitted and wrote the first and final drafts of the submitted manuscript. WVC assembled data to be transmitted and performed summary analyses. MFS, ER, LAC, ATL, DLK, and PKG have participated in development of clinical and genetic data. KET, RMP, MFS, and PKG provided input in organizing analyses and in interpretation of results. CIA, WVC, MFS, ER, LAC, RMP, and PKG provided assistance in manuscript preparation. All authors read and approved the final manuscript.
